# Regulation of Polyomavirus Transcription by Viral and Cellular Factors

**DOI:** 10.3390/v12101072

**Published:** 2020-09-24

**Authors:** June F. Yang, Jianxin You

**Affiliations:** Department of Microbiology, Perelman School of Medicine, University of Pennsylvania, Philadelphia, PA 19104, USA; june.yang@pennmedicine.upenn.edu

**Keywords:** polyomavirus, transcription, tropism, *cis*-acting regulatory elements, cell type-specific transcription factors, epigenetic modifications

## Abstract

Polyomavirus infection is widespread in the human population. This family of viruses normally maintains latent infection within the host cell but can cause a range of human pathologies, especially in immunocompromised individuals. Among several known pathogenic human polyomaviruses, JC polyomavirus (JCPyV) has the potential to cause the demyelinating disease progressive multifocal leukoencephalopathy (PML); BK polyomavirus (BKPyV) can cause nephropathy in kidney transplant recipients, and Merkel cell polyomavirus (MCPyV) is associated with a highly aggressive form of skin cancer, Merkel cell carcinoma (MCC). While the mechanisms by which these viruses give rise to the relevant diseases are not well understood, it is clear that the control of gene expression in each polyomavirus plays an important role in determining the infectious tropism of the virus as well as their potential to promote disease progression. In this review, we discuss the mechanisms governing the transcriptional regulation of these pathogenic human polyomaviruses in addition to the best-studied simian vacuolating virus 40 (SV40). We highlight the roles of viral *cis*-acting DNA elements, encoded proteins and miRNAs that control the viral gene expression. We will also underline the cellular transcription factors and epigenetic modifications that regulate the gene expression of these viruses.

## 1. Introduction

Polyomaviruses are a family of nonenveloped small circular dsDNA viruses that infect a variety of host species, including humans and other primates. The human polyomaviruses JCPyV, BKPyV, and MCPyV are known to widely infect the human population, but in rare cases, cause severe diseases [1]. In immunosuppressed individuals, JCPyV can reactivate from latency to cause the demyelinating disease PML, a fatal pathology of the central nervous system (CNS) [2]. BKPyV is shed in the urine of immunocompetent individuals but in states of immunosuppression is associated with nephropathy, a leading cause of kidney transplant failure [3]. In immunocompromised individuals and the elderly, MCPyV integrates into its host cell genome to cause Merkel cell carcinoma (MCC), a highly aggressive and fatal skin cancer with a steadily increasing incidence rate in recent years [1,4]. For these viruses, the specific molecular events causing asymptomatic viral infection to progress into the relevant diseases have yet to be elucidated.

**Figure 1:**
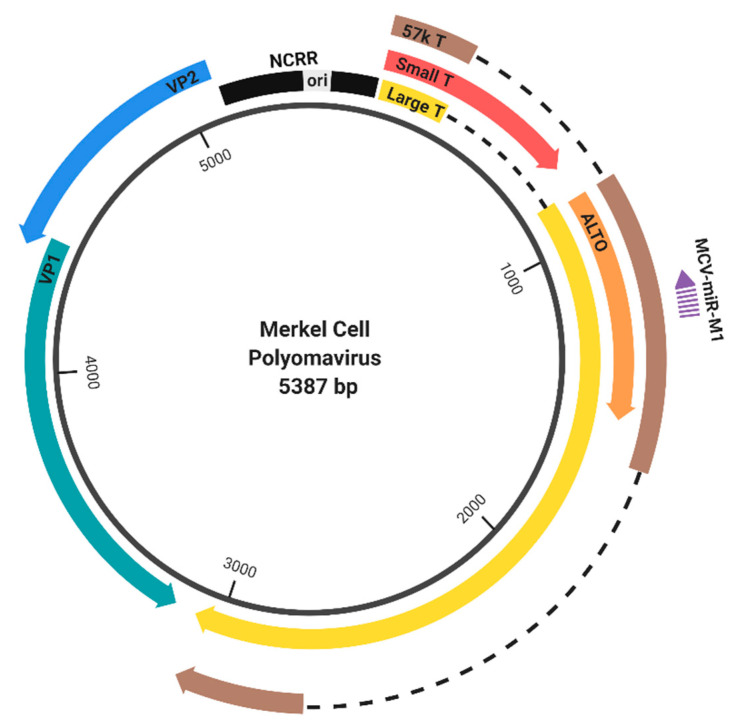
Genome organization of Merkel cell polyomavirus (MCPyV). The MCPyV genome is divided into the early (right) and late (left) region by the noncoding regulatory region (NCRR) [4]. The early region encodes large tumor antigen (LT), small tumor antigen (sT), and the 57 kT antigen by differential splicing, as well as the protein encoded by an Alternate frame of the LT open reading frame (ALTO) [7,8]. The late region encodes the capsid proteins, VP1 and VP2. Gene expression is regulated by NCRR, which contains the origin of replication (ori) and the promoters that drive early and late gene expression. MCPyV also encodes a microRNA (MCV-miR-M1), which targets early transcripts [9].

Members of the polyomaviridae family share the same general genomic structure (Figure 1, depicting MCPyV as an example). The circular genome is divided into the early and late regions, based on the temporal regulation of how these genes are expressed during infection. The early gene locus encodes the tumor (T) antigens, which initiate viral replication and interact with host factors to manipulate the cell cycle. The late gene locus encodes the Viral Proteins (VPs), which are structural proteins needed to assemble the viral capsid. The late regions of SV40, JCPyV, and BKPyV also encode a small regulatory protein called agnoprotein [5]. Transcription of the early and late genes occurs in a bidirectional manner from the noncoding regulatory region (NCRR), also referred to as the noncoding control region (NCCR), which contains *cis*-regulatory elements as well as the origin of replication [4,6] (Figure 1).

The polyomaviruses typically have a very narrow host range and a restricted cell type tropism; in particular, the in vitro tropism of JCPyV is restricted to human glial cells, while MCPyV only productively infects human dermal fibroblasts (HDFs) [1,10,11]. Polyomavirus gene transcription appears to be a key determinant for their viral tropism as well as their pathogenic potential [10,12], highlighting the importance of studying the underlying mechanisms. In this review, we discuss the current understanding of how JCPyV, BKPyV, MCPyV, as well as the well-studied SV40, transcribe the viral genes to contribute to the viral life cycle and associated human diseases. We will review how the viral *cis*-acting regulatory elements, virus-encoded proteins and miRNAs, cell type-specific transcription factors, and epigenetic modifications regulate viral transcription.

## 2. Viral Noncoding Regulatory Region

**Figure 2:**
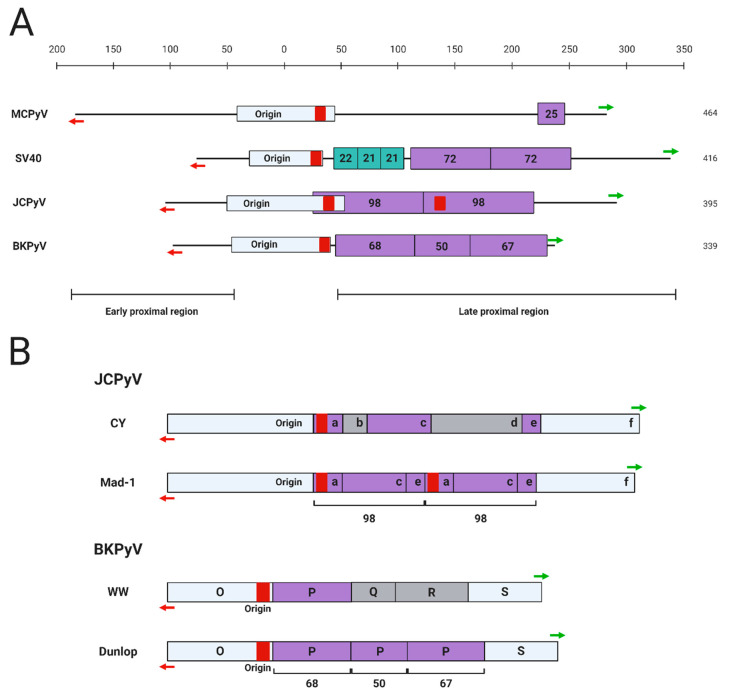
Structure of primate polyomavirus NCRRs. (**A**) The full NCRRs of MCPyV (R17b, GenBank accession no. NC_010277.2), SV40 (776, GenBank accession no. NC_001669.1), JCPyV (Mad-1, GenBank accession no. NC_001699.1), and BKPyV (Dunlop, GenBank accession no. NC_001538.1) are shown. The total nucleotide length of each NCRR is indicated on the right. The origin of replication for each polyomavirus contains a poly A/T tract (red bar), which is duplicated in JCPyV Mad-1. The directions of early and late transcription are shown as red and green arrows, respectively. Enhancer-associated tandem repeat elements in SV40, JCPyV Mad-1, and BKPyV Dunlop are shown in purple. The 25-base pair region of MCPyV duplicated in the Japanese strains (GenBank accession no. LC348865.1) is also highlighted in purple. The SV40 promoter-associated 21-bp tandem repeat elements are marked in turquoise. The approximate early and late proximal regions are indicated. (**B**) The poly A/T tract is indicated as a red bar, and directions of early and late transcription are shown as red and green arrows, respectively. NCRR regions conserved between the prototypical and archetypal strains are shown in purple; regions present in only the archetypal NCRR are shown in gray. The JCPyV archetype (CY, GenBank accession no. AB038249.1) NCRR consists of an early proximal region containing the origin followed by regions a, b, c, d, e, and f. The JCPyV prototype Mad-1 NCRR is arranged as Origin-a-c-e-a-c-e-f [18]. The BKPyV archetype (WW, GenBank accession no. AB211371.1) NCRR consists of an O region (containing the origin), followed by P, Q, R, and S regions. The BKPyV prototype Dunlop NCRR has an O-P-P-P-S arrangement, in which the P regions are imperfectly duplicated [19,20,21]. Tandem repeat enhancer elements in the prototypical strains are also indicated.

For polyomaviruses, the transcriptional control exerted by *cis*-acting regulatory elements such as promoters, enhancers, and silencers is an important factor contributing to the virus’ ability to complete its life cycle in the host cell [12,13,14,15]. This is especially true for MCPyV and JCPyV, each of which maintain a highly restrictive in vitro host range limited to human and chimpanzee dermal fibroblasts and human glial cells, respectively [11,13]. The NCRR region of the primate polyomaviruses contains a core origin region that includes a poly A/T tract essential for the initial unwinding of the viral DNA during replication [16,17] (Figure 2). Overall, however, the sequence and structure of the NCRR are highly varied among the different polyomaviruses (Figure 2A). Specific strains of each polyomavirus also contain rearrangements of the NCRR structure, leading to the categorization of certain polyomavirus strains as “archetypal” or “prototypical” based on the presence of certain *cis*-acting elements in the NCRR (Figure 2B). 

Several significant studies have been devoted to dissecting the *cis*-acting elements controlling the transcription of SV40 since its discovery in the 1960s. In 1981, Benoist and Chambon discovered that two 72-base pair (bp) tandem repeat motifs, now recognized as the enhancer element, within the late proximal region of the SV40 NCRR are essential for early viral transcription [22] (Figure 2A). It was subsequently found that the early promoter region is located within a group of 21-bp tandem repeats of the NCRR [23] (Figure 2A). Interestingly, transcription of SV40 early genes is regulated differently during distinct phases of viral infection. Early transcription is initiated from a site downstream of the origin of replication during early infection, and upstream of the origin of replication during late infection [24]. In addition, the early transcription driven by the downstream sites is selectively enhanced by the 72-bp tandem repeat region [24] (Figure 2A).

JCPyV, BKPyV, and SV40 share a significant degree of homology in their viral gene coding regions and yet are only 40% homologous in their NCRRs [12]. Since each of these viruses has a distinct pathogenic potential, it was hypothesized that the activities of their respective viral enhancers and promoters are responsible for the unique pathologies induced by each virus. Transgenic mice expressing SV40 tumor antigens under regulation of the JCPyV NCRR develop JCPyV-associated pathologies such as hypomyelination and neural tumors, while mice expressing JCPyV genes from the SV40 NCRR develop SV40-associated choroid plexus tumors, suggesting that viral *cis*-regulatory elements not only determine tropism, but also specify disease pathogenicity [12].

The importance of the enhancer element during a successful polyomavirus infection became more apparent when different strains of JCPyV were examined. The prototypical Mad-1 strain of JCPyV, originally isolated from a PML patient, is the most studied variant of the virus [25]. Similar to the 72-bp tandem repeats in the SV40 NCRR, the 98-bp tandem repeats carried by JCPyV Mad-1 strain in the late proximal region of the NCRR enhance viral transcription [26] (Figure 2). However, unlike the SV40 72-bp repeat sequence, which enhances reporter expression in several different cell types, the JCPyV 98-bp repeat only specifically increases viral gene expression in human glial cells [26]. In contrast, an archetypal JCPyV strain isolated from the urine of healthy individuals lacks the enhancer repeats found in Mad-1 [25] (Figure 2B). The archetypal strains of JCPyV constitute the infectious form of the virus, while Mad-1 and similar prototypical strains cause PML, further implicating the role of the enhancer element in pathogenesis [27,28]. Additional rearrangements in the viral NCRR are implicated in the development of the pathogenic strains of this virus. A significant percentage of JCPyV archetypal isolates have short deletions or duplications in the regulatory region, suggesting that this region of the infectious strains is quite unstable [27,28]. Subsequent genomic rearrangements of the archetypal NCRR are therefore hypothesized to give rise to the PML-causing strains, such as Mad-1, that carry the 98-bp tandem repeats. Other, less extensively studied JCPyV NCRR variants exist as well. Many strains of JCPyV isolated from PML patients were generally found to have rearrangements in their NCRRs relative to Mad-1 [29,30]. Additionally, another regulatory region downstream from the enhancer has been identified in the NCRR of JCPyV. JCPyV NCRR reporters harboring a natural deletion in this late gene-adjacent region demonstrate increased viral late gene expression in glial cells compared to the archetype NCRR, indicating a “silencer” function of this region in controlling cell type-specific gene expression [18].

Similar to JCPyV, BKPyV can be divided into prototype and archetype strains based on the presence of transcriptional enhancer repeat elements in the NCRR. The Dunlop strain isolated from a kidney transplant recipient with ureteral stenosis is the most-studied prototypical strain of the virus [31]. The Dunlop strain has three imperfect 68-bp tandem repeat motifs within the NCRR, which act as the enhancer element to promote viral transcription by recruiting host transcription factors [32,33,34] (Figure 2). The archetypal BKPyV-WW, an infectious strain isolated from the urine of immunocompetent individuals, only carries a single 68-bp motif [35] (Figure 2B). This strain has approximately half the enhancer activity of the prototype strain, demonstrating an additive effect of these motifs on transcriptional regulation as well as the impact of NCRR rearrangement on viral infectious activity [35]. Indeed, compared to the archetypal strain, most BKPyV strains isolated from kidney transplant recipients with rearranged NCRRs showed higher viral gene expression and extracellular viral loads [36]. 

Similar to JCPyV, rearrangements in the BKPyV NCRR are suggested to contribute to the formation of the prototypical strains of the virus. It is possible that the archetypal BKPyV NCRR is unstable, allowing rearrangements to arise upon propagation of the virus in infected cells. This notion is supported by the observation that the BKPyV-WW strain carrying only one 68 bp element was isolated and identified directly from the urine of an immunocompetent host, whereas the tandem repeats characteristic of prototypical or rearranged strains appeared only after the BKPyV strains have been passaged in cell culture [37]. Acquisition of mutations in the NCRR of passaged BKPyV was later confirmed using an in vitro BKPyV propagation system, which showed that NCRR variants arose after prolonged propagation of the virus in human endothelial cells [38]. 

Rearrangements of the BKPyV NCRR relative to its archetype strain are associated with more extensive pathogenicity. In vitro, deletion of one of the three 68-bp elements from the prototypical BKPyV strain decreases virus plaque sizes in HEK cultures but increases transformation efficiency of rat cells by the virus [39]. Since this effect is further increased when two of the tandem repeat elements were deleted, it suggests that changes to the transcriptional regulation of the virus can also control the switch between viral propagation and host pathogenesis [39]. Though it appears that rearrangements of the BKPyV NCRR contributed to the switch, the specific mechanisms underlying this change remain poorly understood. Rearranged-NCRR BKPyV strains are also more commonly seen in HIV-1 positive patients, have been detected in a large proportion of BKPyV-associated nephropathy patients, and are associated with more severe disease [3,40,41,42]. BKPyV NCRR rearrangements detected in nephropathy patients also tend to cause nucleotide substitutions, deletions, or duplications of putative transcription factor binding sites in the genome, indicating a role of viral gene transcription in BKPyV-associated disease [34]. 

The MCPyV NCRR is able to drive reporter expression in a number of cancer cell lines and HEK 293 cells, in addition to HDFs that can support productive MCPyV transcription [10,43,44,45]. However, the NCRR of MCPyV shares little homology with that of SV40, JCPyV, and BKPyV, and does not carry any conserved tandem repeats (Figure 2). An analysis of MCPyV-positive MCC cases reveals that MCPyV genomes integrate into the host DNA by “breaking” at sites throughout the viral genome [8,46]. Despite the randomness of the MCPyV integration events, the integrated viral genomes often retain an intact NCRR [46], suggesting that the MCPyV NCRR likely contains the key elements needed for driving viral gene expression in both infected and transformed cells. However, the lack of tandem repeats begs the question of which regions of the MCPyV NCRR are important for driving viral transcription.

Strain-specific NCRR variants of MCPyV also exist. Phylogenetic analyses of global MCPyV strains support the existence of ethnic- or geographic area-associated clades, specifically for North America/Europe, Africa, Oceania, South America, and Asia [47,48,49]. Interestingly, a tandem repeat caused by a 25-bp insertion was identified in the late proximal region of the NCRR found in the Japanese MCPyV strain [50] (Figure 2). However, the functional consequence of the 25-bp insertion remains to be studied. 

Schmidt et al. have determined that inserting additional duplications of the SV40, JCPyV, and BKPyV enhancer elements into their genomes significantly increase viral early transcription and replicative activity during an in vitro infection. Insertion of a “strong” enhancer such as that of cytomegalovirus (CMV) also significantly increases the infectious capacity of the virus, suggesting that the activity of enhancer elements have a profound impact on viral infectivity. As discussed by the authors, similar effects may be seen if enhancer recombination occurs during a co-infection in an in vivo immunocompromised setting, making organ transplant recipients an intriguing population to investigate further [14]. However, no enhancer element has been identified for MCPyV. Nevertheless, inserting an SV40 enhancer into the MCPyV NCRR allows the chimeric virus to infect and express early viral proteins in rat fibroblasts, which are normally nonpermissive to MCPyV infection [13]. This observation suggests that modulation of MCPyV enhancers may broaden its tropism. Further studies of the important *cis*-regulatory elements within the genome of MCPyV are therefore needed in order to fully elucidate the mechanisms controlling its transcription during viral infection and the development of associated skin cancers.

## 3. Viral Proteins

The temporal control of polyomavirus transcription is tightly regulated not only by viral genomic elements but also by the proteins encoded by the viral genome. The LT protein in particular is involved in the transcriptional control of several primate polyomaviruses. In SV40, LT’s viral transcriptional function is primarily to inhibit early transcription in favor of the late transcription [51,52,53,54]. SV40 LT directly interacts with the transcription factor AP-2 to prevent its sequence-specific binding to the viral DNA [55]. SV40 LT itself also interacts with the viral DNA at two specific sites at the origin of replication to inhibit the initiation of early transcription, both in vitro and in vivo [53,54]. In addition to blocking early transcription, LT also acts as a transcription factor to induce late gene expression by binding to the viral NCRR [51,52]. Together, these findings suggest a model of SV40 transcriptional regulation in which early transcription and replication are first favored due to strong promoter activity, and as replicated viral genomes and LT build up, the latter promotes late transcription while repressing early gene expression [56]. Late proteins also play a role in this controlled transcriptional program; the transcription factor Sp1 recruits SV40 VP3 to the viral DNA, where it represses early transcription during the late stage of infection preceding virion assembly [57].

Not only do JCPyV proteins contribute to temporal control of virus transcription, but they also restrict its tropism. Both early and late JCPyV gene expression is restricted only to glial cells. However, expression of either JCPyV or SV40 LT stimulates late gene reporter expression in both glial and non-glial cells, demonstrating that as a viral transcription factor LT has the potential to expand JCPyV tropism [58]. Studies by Safak et al. later showed that JCPyV LT also has inhibitory effects on early transcription, which are alleviated by interaction of the viral protein with the cellular transcription factor YB-1; the two proteins also interact to synergistically induce late transcription [59]. The group went on to further delineate the role of agnoprotein in suppressing early viral transcription through directly interacting with LT and YB-1 [60,61]. Together, their findings suggest a mechanism by which JCPyV transcription is switched to favor late gene expression after the early transcription stage has passed.

The specific roles of MCPyV and BKPyV proteins in viral transcription have not been fully elucidated, although existing evidence suggests that autoregulation of gene expression still occurs. MCPyV LT, for example, induces early viral gene expression in an NCRR reporter system [62]. In addition, the protein coding regions of MCPyV are highly homologous to those of murine polyomavirus, which encodes VP1 to positively modulate its early transcription [4,63]. The coding regions of BKPyV are similarly homologous to the same regions in SV40 and JCPyV [64]. This degree of homology may therefore preserve functional domains among MCPyV and BKPyV proteins, allowing them to fulfill similar functions as those of murine polyomavirus, SV40, and JCPyV in controlling viral transcription. 

Interestingly, proteins encoded by other viruses such as HIV also regulate polyomavirus gene expression. JCPyV, BKPyV, and MCPyV are more prone to cause disease in HIV/AIDS patients [2,3,10]. Besides the general impact of HIV-induced immune suppression in encouraging polyomavirus infection and pathologies, several lines of evidence suggest an additional role of HIV-encoded proteins in directly stimulating polyomavirus transcription. HIV-1 Tat dramatically stimulates BKPyV transcription by inducing the binding of the p65 subunit of NF-κB to the early promoter [65]. JCPyV transcription is also stimulated by HIV-1 Tat, which recruits other cellular proteins to the NCRR; an effect commonly observed amongst all HIV-1 clades [2]. Therefore, HIV-1 may have direct molecular effects on polyomavirus transcription in addition to its function in suppressing the host immunity.

## 4. MicroRNAs

Polyomavirus-encoded miRNAs negatively auto-regulate viral gene expression to control the infectious life cycle. Sullivan and colleagues discovered the specific roles of these miRNAs in each virus, which appear to be evolutionarily conserved [9,66,67]. The SV40 miRNA accumulates late in viral infection and targets early viral transcripts for cleavage [67]. Interestingly, while SV40 early gene expression is downregulated by the viral miRNAs, the infectious virus yield is not affected by the depleting LT protein levels [67]. Cells infected by a miRNA-deficient mutant are more sensitive to lysis by cytotoxic T cells and trigger more inflammatory cytokine signaling [67], suggesting that the viral miRNA may have additional function in immune evasion. JCPyV, BKPyV, and MCPyV also encode miRNAs that recognize and degrade early viral transcripts [9,66]. JCPyV miRNAs are also detectable in the brains of PML patients, indicating their additional role in pathogenesis [66]. In addition to its ability to cause the degradation of early viral gene products, the MCPyV-encoded miRNA contributes to the establishment of long-term episomal persistence of the virus within its host cell. Thus, miRNA-mediated degradation and regulation of viral gene expression may represent a strategy for minimizing MCPyV infection activity for achieving long-term asymptomatic persistence [68]. In addition to infected cells, low levels of MCPyV miRNA have also been detected in nearly 50% of MCPyV-positive MCC samples [69]. In silico analysis indicates that the miRNA expressed in MCCs may target cellular genes involved in antigen presentation and immunoproteasome activation [69]. The MCPyV miRNA may therefore play a similar role in promoting immune evasion as observed with its SV40 counterpart.

## 5. Cell Type-Specific Transcription Factors

Host transcription factors can bind to the *cis*-regulatory elements present within the NCRR and act as activators or repressors of viral gene expression. The presence or absence of these transcription factors within the host cell therefore determines whether expression of the viral genes can occur, and whether an infection can be successfully established within a specific cell type. This has been demonstrated by studies of JCPyV and MCPyV, which revealed that cell type-specific factors are crucial for the viruses to establish infection in their respective host cell [10,11,70]. 

Early experiments established that JCPyV tropism is restricted in glial cells at the level of T antigen expression—the viral promoter and enhancer are only active in glial cells, but viral replication can occur in multiple nonpermissive cell types as long as the SV40 or JCPyV T antigen is present [11]. Expression of the JCPyV T antigen, which occurs specifically in glial cells, is therefore the barrier for the virus to complete the infectious cycle [11]. Heterokaryon experiments showed that T antigen expression is lost in JCPyV-transformed cells upon fusion with JCPyV-nonpermissive cells, indicating that the latter contain factors capable of repressing viral early transcription [15]. Corroborating these historical studies, Ferenczy et al. examined multiple subsets of immortalized human fetal brain cell lines that are derived from the same original culture but support JCPyV infection to different degrees [70]. Their microarray analysis revealed that these closely related cell lines harbored differences within their transcriptomes, and that the transcription factors known to inhibit JCPyV transcription are expressed at high levels in the more resistant cell lines [70]. These findings suggest that cell type-specific expression of transcription activating and/or repressive factors may play a key role in controlling JCPyV tropism. 

A similarly restricted tropism for MCPyV was revealed in 2016 by Liu and colleagues [10]. After examining all the cell types isolated from human skin, it was established that MCPyV is capable of entering multiple cell types, but only expresses viral genes within dermal fibroblasts [10]. Differences in the expression of certain transcription factors in permissive or nonpermissive cell types within the skin likely contribute to the virus tropism. Similar to JCPyV, MCPyV genome can also replicate in nonpermissive cells when its LT is overexpressed [71]. 

Several NCRR-binding transcription factors have been identified for SV40, JCPyV, and BKPyV. By engaging the viral NCRRs, these transcription factors have demonstrated the potential to up- or down-regulate viral gene expression in different viruses. Sp1, identified by Dynan and Tjian in 1983, was among the first factors found to regulate SV40 transcription by binding to the 21-bp tandem repeat region of the SV40 promoter [72]. A total of six Sp1 binding sites were subsequently identified within the SV40 promoter region; three of the sites are engaged by Sp1 to mediate early transcription, while Sp1 binding to the other three controls late transcription [73]. SV40 infection also leads to a tenfold increase of Sp1 expression within the cell, which is attributed to the production of the viral T antigen, suggesting a positive feedback loop in controlling Sp1 and early viral gene expression [74]. The related factor Sp2 is a general transcription factor important for stimulating transcription from the promoters of SV40 and adenovirus, among others. Unlike Sp2, Sp1 selectively upregulates SV40 transcription but inhibits expression from the adenovirus promoter, serving as an example of the cellular factors that can control viral transcription specificity [75]. LSF, AP-1, and AP-4 are additional transcription factors that stimulate late SV40 gene expression. LSF binds the SV40 NCRR upstream of the late transcription initiation site to induce late transcription, while AP-1 and AP-4 recognize adjacent sites in the SV40 enhancer element to cooperatively stimulate late viral gene expression [76,77]. Interestingly, AP-1 also has a repressive function on late transcription initiating from a rarely used start site in vivo, demonstrating its function as both a positive and negative regulator that controls the temporal expression of viral genes [76]. Another related transcription factor, AP-2, was also identified as an SV40 enhancer binding protein that may stimulate viral transcription through functional interaction with Sp1 [55]. 

Several cellular factors that regulate JCPyV transcription have been discovered since the 1990s; the readers are referred to a review containing a visual overview of the binding sites of these transcription factors in the viral NCRR [78]. Tst-1, YB-1, c-Jun, NF-1, C/EBPβ, Spi-B, SRSF1 (also known as SF2/ASF) and the DNA- and RNA-binding factor Pur-α are among the key cellular factors found to bind the 98-bp repeat enhancer elements within the JCPyV NCRR [79,80,81,82,83,84]. Tst-1 stimulates transcription of both JCPyV early and late genes via its DNA-binding activity [79]. YB-1 acts together with the NF-κB subunits p65, p50, and p52 to regulate early and late viral transcription [80]. Overexpression of c-Jun and c-Fos, which dimerize to form the factor AP-1, has an additive inducing effect on both early and late JCPyV gene expression [81]. On the other hand, CEBP/β represses JCPyV transcription upon binding to a site adjacent to the NF-κB binding site in the early proximal region of the NCRR [85]. Romagnoli et al. also detected a complex of NF-κB/p65, CEBP/β, and JCPyV DNA in glioblastoma cells, providing evidence to support that the opposing activities of p65 and CEBP/β determine whether or not JCPyV reactivates to cause PML [85]. Additionally, SRSF1 acts as a potent repressor of JCPyV gene expression by binding to the viral promoter. In order to circumvent this viral transcriptional repression, both the viral LT protein and Pur-α inhibit SRSF1 at the transcriptional level [82,83]. Pur-α also represses SRSF1 at the protein level, and is able to induce the viral early promoter after binding to LT [82,83]. Interestingly, SRSF1 also suppresses LT expression in JCPyV-transformed cell lines [84]. Repression of SRSF1 in the transformed cells leads to cell growth and proliferation, supporting its role in blocking JCPyV-induced cellular transformation [84].

Certain cell type-specific JCPyV transcription factors are crucial in determining the host cell permissiveness to infection. Tst-1, the key transcription factor controlling JCPyV transcription, is specifically expressed in myelinating glial cells and therefore is likely a major determinant of JCPyV tropism [79]. Additionally, the JCPyV NCRR binding site for the activating factor c-Jun is adjacent to that of NF-1, suggesting that the two factors may directly interact with each other to control viral transcription. Notably, many genes expressed in the central and peripheral nervous systems also contain adjacent binding sites for NF-1 and an additional transcription-activating factor upstream of the mRNA start site [86]. This finding suggests that the expression of JCPyV genes shares a common regulatory pathway with other cellular genes in the nervous system, and that this could be a strategy by which the virus adapts to specifically infect glial cells [86]. Corroborating this finding, Kumar et al. discovered that mutating the two NF-1 sites in the JCPyV enhancer region caused a significant reduction of JCPyV early gene expression specifically in glial cells [87]. Exposure of glial cells to pro-inflammatory cytokines, which normally occurs in the CNS of HIV/AIDS patients, further induces NF-1 binding and early transcriptional activation from the JCPyV NCRR, highlighting a potential mechanism underlying the higher PML incidences among HIV/AIDS patients [88]. Another transcription factor, Spi-B, was found by Marshall et al. to regulate JCPyV transcription in both a cell- and strain-specific manner. Spi-B has different binding sites in the prototypical and archetypal NCRRs, but only bound and stimulated transcription from the prototypical NCRR [89]. Exogenous expression of this factor in nonpermissive cells also resulted in JCPyV LT expression, indicating Spi-B’s role in conferring cell type specificity to JCPyV gene expression in PML-associated strains [89]. Another NCRR-binding complex consisting of the RNA helicase DDX1 and the cleavage stimulation factor CtsF was isolated from a JCPyV-susceptible neuroblastoma cell line [90]. DDX1 is specifically expressed in neuroblastoma cells and binds directly to the JCPyV NCRR [90]. While DDX1 overexpression induces both early and late viral gene expression, its knockdown suppresses JCPyV infection in the permissive neuroblastoma cell line [91]. Together, these observations suggest that DDX1 is also involved in defining JCPyV host cell specificity [91]. 

Research into the regulation of BKPyV gene expression has revealed complex relationships among the transcription factors that bind to the viral NCRR; an overview of the binding sites for these factors is included in the following reference [92]. Early work by Shivakumar and Das discovered that p53 binds to a site in the BKPyV NCRR to transactivate gene expression in a reporter system containing the isolated p53 binding site, but represses BKPyV gene expression when binding to the whole viral promoter. Additionally, Sp-1 and NF-1 were purified in a complex with p53, implying that specific interactions among these two proteins and p53 modulate its effect on BKPyV transcription in infected cells [93]. In line with this finding, NF-1 has separately been found to bind to the late promoter initiation site of the archetypal BKPyV strain to repress viral gene expression [94]. On the other hand, the p65 subunit of NF-κB was found to transactivate the BKPyV early promoter, whereas C/EBPβ, which binds an adjacent site on the NCRR, can interact and synergize with p65 to induce viral gene expression [95]. More recently, Jordan et al. discovered that NFAT binds to three sites in the BKPyV NCRR and is capable of both activating or repressing viral gene expression through cooperation with c-Jun or p65, which also bind to their respective sites in the NCRR [96]. To further examine the role of transcription factors in regulating BKPyV gene expression, Bethge et al. mutated a total of 28 transcription factor binding sites in the archetype BKPyV NCRR and analyzed the impact on viral promoter activity [97]. They found that these transcription factors have a wide range of effects on viral transcription that can be divided into three classes. Inactivation of one of the Sp1 or Ets1 sites near the late transcription start site increases early transcription of the mutant virus to levels observed with rearranged-NCRR BKPyV; mutations in the NF1, YY1, and p53 sites moderately increases early gene expression; whereas mutating Sp1 sites near the early transcription start site abolishes both early and late gene expression [97]. The three different groups of mutations were also associated with significantly increased, moderate, and no viral replication, respectively. Therefore, the location of Sp1 sites, combined with specific transcription factors expressed in the host cell, appears to be crucial for controlling not only the balance of early and late BKPyV gene expression, but also its replicative capacity [97]. 

It should also be noted that many of the SV40 transcription factors identified in early studies by the Tjian lab were found in HeLa cell extracts. However, the SV40 enhancer is active in both HeLa and lymphoid B cells. A comparison of extracted proteins from these two cell types revealed that some of the SV40 enhancer-binding proteins are HeLa- or B lymphoid-specific [98], revealing a host cell factor-dependent mechanism in controlling SV40 gene expression.

A number of well-known transcription factors such as NF-κB, Sp1, Ets1, and NF-1 have been predicted to bind to the MCPyV NCRR in silico [62], though currently none have been experimentally shown to mediate the transcription of this virus. While MCPyV is able to enter many different types of cells in the human skin, viral gene expression is only detected in HDFs [10]. Additionally, dermal fibroblasts from a variety of common model animals only support viral entry, but not viral gene expression [13]. These observations suggest that, similar to the mechanisms that control the restricted tropism of JCPyV, transcription factors needed to support MCPyV gene expression may be highly expressed in HDFs and not in nonpermissive cell types elsewhere in the skin or in different animals. In addition, as discussed below, a distinct set of epigenetic modifications may be involved in the transcription regulation of the virus in different host cells. Finally, activation of the MAPK/ERK pathway is crucial for stimulating MCPyV transcription [10], while inhibition of MEK1 and MEK2 abolishes viral transcription. During in vitro infection of HDFs, treating the cells with FBS, which induces the MAPK/ERK pathway [99], stimulates viral transcription in a dose-dependent manner [10]. Therefore, it is also possible that the transcription factors stimulated by the MAPK/ERK pathway may directly regulate MCPyV transcription.

## 6. Epigenetic Modifications

The small, circular genomes of polyomaviruses are packaged together with cellular histones into a viral episome [100,101,102,103]. Epigenetic modifications, including histone lysine acetylation and deacetylation, histone lysine methylation, DNA methylation, and nucleosome positioning are therefore implicated in regulating viral gene expression [104,105,106]. Acetylation of Histone H3 and H4 and methylation on H3K4/H3K36 are associated with transcriptional activation, whereas tri-methylation of H3K9 and H3K27 is usually linked to transcriptional repression. In addition, DNA methylation is associated with transcriptional silencing through a number of pathways including recruitment of histone deacetylases and blocking of transcription factor binding [105,106]. Finally, the positioning of nucleosomes along the chromatin also controls the ability of the transcriptional machinery to access the viral DNA [105]. 

Largely due to the efforts of the Milavetz lab, the epigenetic regulation of SV40 is, thus far, the most studied among the polyomaviruses. The readers are referred to a recent review by Balakrishnan and Milavetz for findings on the epigenetic regulation of polyomaviruses made by their lab and others [105]. Histone acetylation of SV40 episomes is a major mechanism by which viral transcription is regulated. In early infection, SV40 episomes are hyperacetylated on either histone H4 or on both histones H3 and H4; the H4-only subclass is subsequently deacetylated later in infection, demonstrating two different modes of epigenetic regulation of viral gene expression [107]. During viral transcription, hyperacetylation and deacetylation of the SV40 episome are mediated by the histone acetylase p300 and an unknown histone deacetylase (HDAC) while RNAPII simultaneously transcribes the viral genome [100]. As observed with the cellular genome, the epigenetic memory of SV40 DNA is also preserved in packaged virions and carried into subsequent infections [108]. 

The epigenetic regulation of other primate polyomaviruses is less well studied, but early evidence suggest that similar mechanisms may control viral transcription. For example, treatment of JCPyV-infected cells with an HDAC inhibitor significantly induces viral transcription [109]. In fact, HDAC inhibitor treatment caused a tenfold higher induction of JCPyV NCRR reporter expression in nonglial vs. glial cells, suggesting that hypoacetylation of the JCPyV NCRR may be a major mechanism by which viral transcription is suppressed in nonpermissive cells [110]. In a recent study on BKPyV, Fang et al. showed that the BKPyV DNA also adopts histones from host cells and forms minichromosomes during the viral life cycle [111]. By combining nanoflow liquid chromatography with tandem mass spectrometry (LC-MS/MS), they identified histone acetylation, methylation, phosphorylation, ubiquitination, formylation as well as several novel post-translational modifications associated with the BKPyV episomes [111]. This global profiling of histone modifications in BKPyV episomes therefore provides the groundwork for future studies on the epigenetic regulation of BKPyV transcription. 

The epigenetic regulation of MCPyV infection remains largely unknown. However, histones are packaged inside the MCPyV virions [101]. After MCPyV genomes are transfected into PFSK-1 cells that support viral gene expression, transactivating histone marks are detected in the NCRR region [68]. Therefore, like other polyomavirus genomes [112], the MCPyV genome is likely packaged into histone-bound episomes carrying epigenetic modifications that can control viral gene expression in infected cells. MCPyV transcription is active only in permissive cells such as HDFs and MCC cells, but silenced in nonpermissive cells such as human foreskin keratinocytes (HFKs) [10]. This observation suggests that the viral genomes may acquire different epigenetic modifications in these cells to support its distinct transcription activities. 

The SV40, JCPyV, and BKPyV genomes have significantly fewer methylatable CpGs in their genomes than is predicted for their genome size [5], suggesting that DNA methylation is not likely to affect viral transcription. Indeed, Graessmann et al. observed that in vitro methylation of SV40 or murine polyomavirus genomes by rat liver methylase did not introduce significant changes in early gene expression, replication, or transformation when the viral genomes were microinjected into live cells; however, in vitro methylation of a single CpG in the late proximal region of the NCRR is associated with reduced VP1 production, indicating that late transcription alone may be affected by DNA methylation [113,114]. Treatment of JCPyV-infected cells with a DNA methyltransferase inhibitor also does not introduce observable effects on viral transcription [109]. In addition, bisulfite sequencing of BKPyV virions and replicating genomes confirmed that none of the CpGs within the BKPyV NCRR or its coding regions are methylated [115]. 

Studies examining the promoters of cellular genes have found that Sp1 binding sites protect nearby CpG islands from de novo methylation [116,117]. The presence of Sp1 binding sites in the NCRRs of all the primate polyomaviruses discussed suggests that this is a potential mechanism that masks the viral genome from DNA methylation [62,72,97,118]. Given that DNA methylation can inhibit the binding of transcription factors such as NF-κB, AP-2, E2F, and c-Myc to DNA, the lack of this epigenetic modification in SV40, JCPyV, and BKPyV may represent an evolutionarily conserved mechanism to prevent the blockage of viral transcription by the host DNA methyltransferases [5,106]. Nevertheless, how DNA methylation could impact MCPyV transcription remains to be investigated. 

## 7. Conclusions and Future Perspectives

Decades of research on SV40 have established it as a model system to study eukaryotic transcription. Research on SV40 and other polyomaviruses has also uncovered viral genomic elements and host cell-specific transcription factors that function together to regulate the viral gene expression. In addition, polyomaviruses have been found to hijack cellular signaling pathways such as the MAPK/ERK signaling cascade [10,119,120] and the NF-κB pathway [80,95,121,122] to support viral transcription. Emerging evidence suggests that the host DNA damage response (DDR) pathways are also exploited by the polyomaviruses to support their gene expression, infection and pathogenic potential [123,124,125,126], though the specific roles of DDR components in these processes remain to be fully elucidated. In recent years, research on the three-dimensional chromosomal topology of several DNA viruses during infection has provided exciting new insight to suggest that viral episomal conformation may also play an important role in controlling viral gene expression, though polyomaviruses have yet to be studied in this context [127,128,129,130]. Therefore, this is an area of interest for future polyomavirus transcription research.

JCPyV-associated PML is among several debilitating neurological complications that commonly affect HIV/AIDS patients [2,29], whereas BKPyV reactivation in immunosuppressed kidney transplant patients can cause nephropathy associated with up to an 80% chance of graft rejection [3,42]. Therefore, studies to further address the molecular mechanisms governing the regulation of JCPyV and BKPyV transcription could facilitate the development of effective targeted therapies against PML and nephropathy, the devastating diseases that continue to pose significant risks to immunocompromised individuals.

Compared to SV40, JCPyV, and BKPyV, the transcriptional regulation of MCPyV is much less understood, though it is clear that transcription of viral genes plays a critical role in both maintaining persistent viral infection and defining its host tropism and oncogenic potential. MCPyV DNA is clonally integrated into the host genome in at least 80% of MCCs [131,132]. In these tumor cells, the MCPyV early promoter drives the expression of the early viral oncogenes, sT and a truncated form of LT, from the integrated viral genome to support cancer cell growth [46,133,134,135]. MCPyV-positive MCCs are addicted to the viral oncoproteins and require their continued expression to survive [8,133,134,135]. MCPyV-driven oncogene expression therefore represents an attractive MCC therapeutic target for molecular intervention. Like the other primate polyomaviruses, expression of MCPyV tumor antigens may be controlled by the promoter and enhancer elements present in the NCRR as well as cell type-specific transcription factors. However, very little is known about the mechanisms that regulate MCPyV transcription in either infected cells or MCPyV-positive MCC cells. Discovery of HDFs as the permissive cells that support MCPyV infection in the human skin reveals a new opportunity to study the *cis*-acting viral DNA elements, the host cellular factors, as well as the epigenetic modifications that regulate MCPyV transcription [10,13,136]. These studies may identify druggable targets for specific blockage of MCPyV oncogene expression and MCC tumor growth, thus offering promising leads for developing novel virus-targeted therapies to treat the highly lethal MCC cancer.
